# Advanced Post-Processing Techniques for Hydrophobic and Flame-Retardant Textiles

**DOI:** 10.3390/polym17202744

**Published:** 2025-10-14

**Authors:** Andrey A. Vodyashkin, Mstislav O. Makeev

**Affiliations:** Bauman Moscow State Technical University (BMSTU), 2-ya Baumanskaya St., 5, p.1, Moscow 105005, Russia

**Keywords:** hydrophobicity, fire retardant, flame-retardant textiles, fibres, fabric surface modifications, functional textiles

## Abstract

Textiles are items of everyday use, and their production occurs in huge quantities worldwide. Providing textile materials with additional protective functions can enhance safety and serve as a foundation for developing personal protective equipment. This review summarises strategies for post-production modification of textile fabrics aimed at obtaining hydrophobic and flame-resistant products. Reported methods for hydrophobization are outlined, including approaches based on surface morphology control and chemical treatment, which have been applied to create moisture-proof textiles. In addition, developments in strategies for producing flame-resistant materials are discussed, with potential applications ranging from specialised protective clothing to the enhancement of everyday garments. In this article, we demonstrate simple and safe methods for producing textiles with a contact angle of approximately 150°. We present approaches, including environmentally friendly ones, that enable the creation of cotton materials with an LOI greater than 35%.

## 1. Introduction

Textiles can improve human comfort and protect against various impacts [1,2,3,4,5]. Everyday textile products can improve overall human comfort and can also be used to create specialised clothing [6,7,8,9,10,11,12]. In recent years, the development of functional textiles has become increasingly important, and chemical agents of various types can be used for these purposes [13,14,15,16,17].

Textile functionalization can address a variety of challenges, such as improving tactile properties, enhancing colorimetric performance, increasing mechanical strength, and creating fabrics capable of protecting against different types of external impacts [18,19,20,21,22,23,24]. Clothing, in particular, can be designed to provide protection from biological agents (bacteria, viruses, etc.), extreme temperatures, and mechanical stress, as well as natural and anthropogenic hazards [25,26,27,28,29,30].

Various substances can be used to functionalize textiles, including both small molecules and polymeric compounds [31,32,33,34,35,36]. In recent years, the use of nano- and micromaterials has been actively developed, enabling not only surface functionalization but also adjustment of fabric morphology [37,38,39,40,41,42,43,44]. Considering the large-scale production of textiles, the use of natural and eco-friendly materials for creating functionalized products has gained particular importance [45,46,47,48,49]. Natural agents as modifiers expand application possibilities and simplify safety requirements in textile manufacturing or modification processes [50,51,52,53,54,55]. Moreover, such agents may be incorporated into formulations designed for personal use, allowing individuals to apply functionalizing compositions at home.

Fabric functionalization can be achieved through multiple approaches [56,57,58,59,60]. A traditional method involves incorporating functional substances during fibre production. This allows for uniform distribution throughout the bulk of the material. However, such approaches often restrict the range of potential applications and can be technologically complex. Simpler strategies include immersing textiles in functionalizing solutions or applying treatments by spraying. These methods can be performed at virtually any stage of textile manufacturing and are also applicable to finished products. Nevertheless, it is important to consider changes in morphological and mechanical properties following modification, since these factors can strongly influence the comfort of clothing use [61,62,63].

This review highlights versatile approaches reported in the literature for the development of hydrophobic and flame-resistant textile materials. Recent functionalization methods with potential for integration into industrial textile production are discussed. In addition, quality control techniques relevant for assessing textile resistance to ignition and combustion are summarised.

## 2. Research Methodology

To prepare this review work, publicly available databases Scopus and Dimensions were used. Initially, key articles that had a significant impact on the textile industry and the field of functionalized materials were analysed. For this, articles with the highest number of views and citations were considered. The aim of this study is to present the most current and promising textile modification methods. For this purpose, we analysed papers found using the queries “hydrophobic textile, hydrophobic fabric, hydrophobic fiber” or “flame-retardant fabric, flame-retardant textile, flame-retardant fiber” in the Scopus and Dimensions databases for the period from 2012 to 2024. Articles demonstrating repetitive approaches to textile modification were excluded from our review. The primary selection criterion for our article was the ability to integrate the modification procedure into current technological procedures. The works were systematised, and the simplest and most promising approaches to functionalization were identified, which are presented in Section 3 and Section 4 of this article.

## 3. Hydrophobisation of Textile Materials

Hydrophobic textiles are garnering significant interest from both industry and academia due to their water-repellent and self-cleaning characteristics. These properties can either be engineered during the manufacturing process or applied to finished goods. As a result, these advanced materials show considerable promise for use in personal protective equipment and everyday outerwear [64,65].

The phenomenon of the hydrophobic effect can be described as follows: water droplets (hydrophilic liquids) in contact with a surface adopt a close spherical shape and roll easily off the surface [66]. In our work, we use the term “hydrophobic materials” for products with a contact angle greater than 90°. We use this parameter as a universal one and apply it to the vast majority of studies. We note that the developed textile surface and micro- and macro-roughness are not taken into account in this method, but it nevertheless allows for a comparative analysis and a comprehensive assessment of the material’s wettability. The effect under discussion has been shown to have several notable consequences. Firstly, it has been demonstrated that the functionality of materials and products made from them can be increased. Secondly, the service life of the materials can be extended. Thirdly, the costs of washing and processing can be reduced [67,68]. As demonstrated in [69,70], the effect can be particularly useful in the context of personal protective equipment and specialised protective clothing.

In order to create hydrophobic materials, it is essential to consider two key parameters: surface roughness [71] and surface chemical composition [72]. The formation of hydrophobic materials, particularly in the context of textile fabrics, is contingent on both micro- and macro-roughness. These characteristics can be engineered through material texturing, the application and fixation of nanostructures, and analogous methodologies [73].

In view of the considerable heterogeneity of the material, its fibre content and the advanced state of its surface, it is a very challenging task—one that is also expensive in financial terms—to create a specific pattern for use in the creation of a hydrophobic surface. The surface chemicals should be selected to provide low surface free energy whilst also ensuring that they have minimal effects on the mechanical properties of the material without altering the tactile sensations. The utilisation of organosilicon is a viable methodology for the formulation of hydrophobic coatings [74,75]. In addition to this, organofluoride reagents [76] and nanoparticle-based solutions [77] have also been demonstrated to be efficacious modalities for the modification of surface properties. In such cases, polymeric substances capable of polymerizing in situ on the surface of textiles or chemically crosslinking (forming a covalent bond) them in order to increase the stability of the coating are usually used for this purpose [78,79].

A comprehensive overview of the development of hydrophobic and superhydrophobic textile materials has been provided in previous works: [80,81,82,83,84,85]. In the following review, the principal methodologies and tenets underpinning the development of hydrophobic textile materials will be expounded upon, with a particular emphasis on recent innovations in the field.

### 3.1. Hydrophobisation of Textile Materials by Chemical Modification

**Figure 1:**
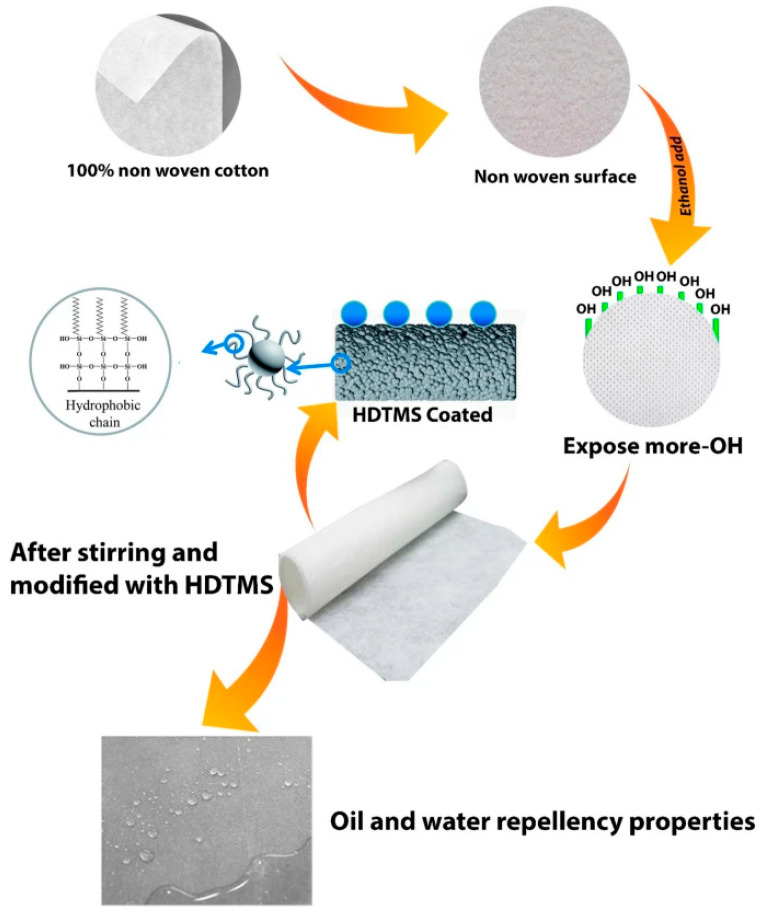
Scheme of HDTMS application on the surface of nonwoven cotton material [86].

Krisna Saha et al. proposed a conventional approach to surface modification using organosilicon. Hexadecyltrimethoxysilane (HDTMS) was utilised as the primary surface modifier. The reagent was applied from an ethanol solution onto non-octane cotton by stirring at elevated temperature in a round bottom flask (Figure 1). It was established that an increase in the concentration of HDTMS in the system resulted in an increase in the cotton material’s marginal wetting angle. The modification was necessitated by the interaction of alkoxysilyl groups of HDTMS and hydroxyl groups of cotton substances, a phenomenon that was confirmed by FTIR and elemental analysis. In order to reduce the cost of the surface modification procedure, the application conditions (mixing time and intensity, temperature, amount of ethanol) were optimised. Consequently, an edge wetting angle of 127.4° was achieved when treated with a system including 2 mL HDTMS, 40 mL ethanol, at 60 °C for 3 h. Such conditions permitted a reduction in the consumption of hydrophobising agent and the treatment time of textile fabrics. It is important to note that a drop of water, even when on the surface for more than 115 min, did not significantly change the marginal wetting angle of the material (from 127° to 120°). The modified coating demonstrated stability even after 15 washings, a crucial consideration for the practical implementation of the method in various sectors of light industry [86]. This paper presents a simple strategy for creating hydrophobic textiles that can be used to modify clothing; however, further study of the mechanical properties and breathability of the final product is required.

In their presentation, Mei Liu et al. set out a method for the surface modification of cellulose nonwoven material using monoisocyanates with different functional groups. In the present study, 3-isocyanatopropyltrimethoxysilane (ISPTMOS), tert-butylisocyanate (TBIS) and m-toluene isocyanate (MTIS) were utilised for the modification of the fabric. The influence of these agents on the hydrophobising properties of the fabric was investigated, with the application being conducted from a hexane solution at an elevated temperature. It was evident that all samples led to a significant increase in the hydrophobicity of the material. However, ISPTMOS was found to provide the highest efficiency when the lowest concentration was used and the edge wetting angle reached 140°. In this study, the dependence of the concentration of the modifying agent on the edge wetting angle of the material was established. Following the modification process, an increase in the material’s roughness was observed, concomitant with a decrease in the average fibre diameter. This phenomenon can be attributed to the heat treatment of the material during the modification process. Conversely, modification with monoisocyanates resulted in a significant decrease in pore diameter, which may lead to a decrease in vapour and air permeability. The tensile strength of the materials was found to be contingent upon the type of modifier employed, as well as its concentration. The TBIS modification did not result in any alteration to the material’s inherent strength. However, the ISPTMOS modification led to an augmentation in strength, while the MTIS modification resulted in a reduction. ISPTMOS caused a significant alteration to the material’s flexibility, while TBIS and MTIS had no impact on this property. Consequently, these substances could be utilised in the production of textile materials intended for clothing applications [87].

Similar modification methods were proposed in the work of Hongchen Liu [88]. The modification process involved a two-stage procedure. Initially, the fabric was treated with tert-butylacetoacetate from a DMFA solution. Subsequently, the material was treated with a combination of stearaldehyde, ammonium acetate and gentamicin sulphate in an ethanol solution. This modification resulted in the material acquiring hydrophobic properties, as well as exhibiting antibacterial and fluorescent properties.

In their seminal paper, Mengwei Dai et al. proposed a novel method for the production of an aqueous dispersion of polyurethane. This innovative material was then used to functionalise the surface of a material composed of 70% polyester and 30% cotton. In this work, a solution method was proposed to produce an aqueous dispersion of polyurethane with terminal hydroxyl groups and modified with polydimethylsiloxane with terminal hydroxyl groups (HPDMS). The textiles were initially subjected to a pre-cleaning process, after which they were immersed in a solution of polyurethane with varying degrees of HPDMS substitution and ultrasonicated. Thereafter, the excess liquid was removed from the surface, and the textiles were cured to minimise coating thickness. The wettability of the textile was found to be contingent upon the extent of HPDMS incorporation into the polyurethane matrix. Attaining a 10% substitution level resulted in an edge angle of 154°, thereby evidencing pronounced hydrophobic characteristics. In doing so, the authors demonstrated the possibility of incorporating carbon nanomaterials into the modifying matrix, which would provide conductive and radio-shielding properties to the textile material [89].

### 3.2. Hydrophobisation of Textiles by Surface Morphology Modification

As proposed by Nives Matjakovič Mlinarić et al., an innovative method of layer-by-layer modification of textile fabrics has been developed, utilising poly(allylamine) hydrochloride in conjunction with zinc nanoparticles. The treatment was carried out on nylon, cotton and polyester, which were subjected to a pre-treatment in an alkaline solution, followed by a thorough washing with water. Thereafter, the materials were immersed in a solution of poly(allylamine hydrochloride) and in a suspension of ZnO nanoparticles, with the addition of further ultrasonic treatment. The procedure was repeated multiple times until three layers of ZnONPs remained on the textile. Thereafter, the textile was washed and dried using warm air. Surface modification resulted in a substantial alteration to the zeta potential of the textile. The zeta potential exhibited a shift from a negative value (as observed in the native textile samples) to a positive value, with polyester demonstrating the most significant potential at 70.8 ± 4.4 mV. The implementation of a single layer of treatment resulted in an increase in the marginal wetting angle (greater than 95°) for nylon and cotton, thereby altering the surface properties of the materials from hydrophilic to hydrophobic. In the case of EDS (Energy-dispersive X-ray spectroscopy), the uniform distribution of zinc particles across the fibres was demonstrated, ensuring homogeneous properties across the entire area of the textile materials. Furthermore, the antibacterial activity of planktonic and adhered *Staphylococcus aureus* bacteria was demonstrated, due to zinc nanoparticles and surface charge [90]. The use of zinc resulted in the limitation of bacterial cell growth.

Cuiying Ye et al. proposed a two-step modification method for polyester plain weave fabrics with a thickness of 343 μm. It is important to note that surface topography can have a key impact on wettability and other functional properties of the material, so the polyester described in this study may not be equivalent to that presented in other studies. However, the functionalization method can be used for a variety of functionalization methods. The modification procedure entailed pretreatment with a NaOH solution for a duration of one hour at a temperature of 60 °C, subsequently followed by a washing step. The fabric was then placed in a suspension of silica nanoparticles for 2 min, after which the fabric was immersed in a solution of 1H,1H,2H,2H,2H-Perfluorodecyltrichlorosilane and poly(vinylidene fluoride-co-hexafluoropropylene) for 2 min. The fabric was then subjected to a drying process at a temperature of 98 °C for a duration of 1 h. This resulted in a material with an edge wetting angle of 157°. In this study, the concentrations of 1H,1H,2H,2H-Perfluorodecyltrichlorosilane and poly(vinylidene fluoride-co-hexafluoropropylene) in the modifying solution were optimised. The surface-modifying layer can be recovered through the process of heat treatment, which involves subjecting the layer to a temperature of 130 °C for a duration of 10 min. The wetting edge angle remained constant after exposure of the fabric to acid and alkali solutions for 50 h and subsequent washing [91].

**Figure 2:**
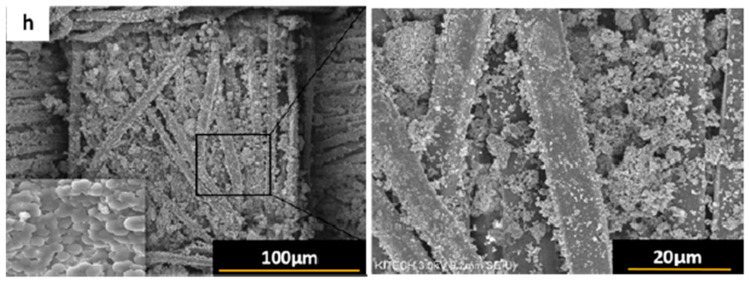
Surface morphology of PET tissue modified with OTS and SiO_2_ [92].

Mahshab Sheraz et al. presented a hydrofibilising system for PET based on hollow silica nanoparticles and octadecyltrichlorosilane (OTS) [92]. The modifying composition was prepared by mixing a solution of OTS in water, hexane and hollow SiO_2_ nanoparticles using a magnetic stirrer. The textile surface was applied by subjecting the textile to an overnight soak in the modifying composition. The modifying composition due to silica nanoparticles had a significant effect on PET surface morphology (Figure 2). The utilisation of OTS solution in isolation yielded an edge wetting angle of PET of 143°, while the incorporation of 2% silica nanoparticles elevated the edge wetting angle to 159°. This augmentation can be attributed to the elevated hydrophobicity of the particles and the concomitant formation of additional surface roughness. The coating was subjected to 20 washing cycles and exhibited negligible change in the marginal angle (up to 153°). The method proposed by the authors has been shown to be highly stable and to be devoid of toxic reagents for surface modification. This allows for its integration into a variety of industrial areas.

In their study, Sukanta Pal and their team of researchers proposed an innovative method for hydrophobising cotton fabric using silver and silane nanoparticles. The modification procedure was executed in a series of stages. The cotton material underwent a series of treatments involving an initial wash in an alkaline solution, followed by exposure to ethanol in an AgNO_3_ solution for a duration of two hours. Subsequent to this, the material was subjected to a drying process, followed by an immersion in a citric acid solution for a period of thirty minutes. The material was then dried once more at a temperature of 70 °C, after which it was soaked in a solution of a hydrolysed silane complex (3-(trimethoxysilyl)propyl methacrylate) (TMSPM) for a duration of two hours. The final step in the process entailed the removal of excess modifying agents and the drying of the fabric. The wetting edge angle exhibited a range from 139° to 149°, contingent upon the concentration of the silane complex. The treated material exhibited hydrophobic properties, rendering it resistant to absorption from milk, tea and other liquids. The adhesive tape test (sticking/unsticking) demonstrated the stability of the coating after 30 cycles, maintaining an edge angle of 140°. Furthermore, the tissue paper and finger abrasion tests demonstrated no substantial impact on the edge wetting angle. The modification method proposed by the authors of this study enabled the utilisation of cotton materials as filtration materials for liquid separation [93]. The integration of silver nanoparticles and complex silane resulted in the creation of a cotton material with a high degree of hydrophobicity. However, this process also led to a substantial alteration in the colouration of the material, from white to brown. This variation in colouration may impose limitations on the method’s applicability in specific contexts. Furthermore, the modification process may also exert a substantial influence on the mechanical properties of the textile.

### 3.3. Hydrophobisation of Textiles with Natural Organic Agents

In their presentation, José Gonçalves et al. set out a method for hydrophobising cotton materials using zein. Zein is a natural protein that is primarily isolated from maize kernels. It has been established that zein possesses an amphiphilic character, which enables the formation of metastable macromolecular micelles within aqueous solutions. The process of hydrophobisation was found to be contingent upon the concentration and method of application of zein to the surface. The most efficacious application method is by way of addition. Samples at a concentration of 50 g/l demonstrated the most rapid time in absorption of water droplets in treated cotton. The authors demonstrated the importance of the ethanol content in the solvent on the edge wetting angle of modified textile materials. The use of SEM (scanning electron microscopy) revealed that zein forms a thin layer on the surface of the material and also fills the voids between the fibres. Concurrently, the treatment of zein resulted in an augmentation of the material’s strength, a development that may positively influence its integration into the industrial sector [94].

**Figure 3:**
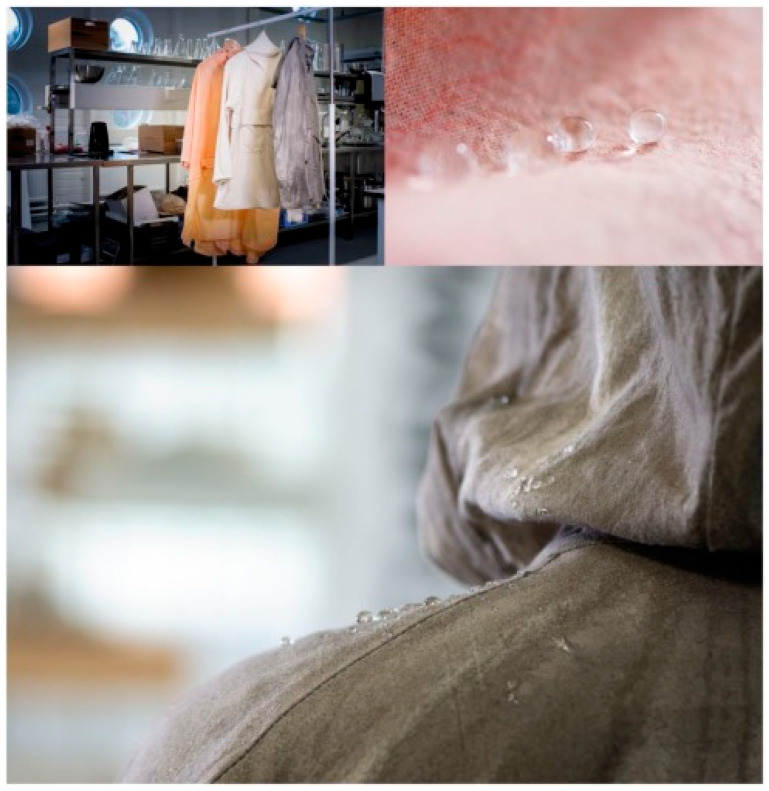
Samples of textile products obtained by industrial spraying method [95].

In their presentation, Nina Forsman et al. set out a method of coating textile fabrics with a layer-by-layer hydrophobising agent. The utilisation of natural agents, including poly-L-lysine (PLL), cationic starch (CS) and wax, has been proposed as a means to modify the properties of the resulting materials. For the purposes of application, the fabrics were immersed in solutions of the modifiers at concentrations of 1 g/L for PLL, 5 g/L for CS, and 10 g/L for CS. Initially, a layer of PLL or CS was applied and subsequently dried at ambient temperature. Thereafter, the material was cured at an elevated temperature for a minimum of 15 min. Subsequently, a hydrophobising agent in the form of wax was applied to the surface. The curing temperature was found to have a significant impact on the edge wetting angle of the textile, with the highest angle being achieved at 70 °C. Concurrently, the coating exhibited a water-repellent property that persisted for a duration exceeding 2.5 h. The incorporation of a single layer of PLL with wax resulted in the attainment of optimal hydrophobic properties. This outcome was replicated by the starch/wax coating, which achieved comparable performance with a mere two layers. It is acknowledged, however, that PLL incurs a relatively high cost, and its integration into industry has the potential to result in a substantial increase in the cost of goods. Notwithstanding the implementation of high molecular weight substances on the surface, there was no decline in vapour permeability of the material in comparison with the untreated textile material. It is important to note that, depending on the type of material, the modification allows different marginal wetting angles to be achieved. However, significant hydrophobisation occurs on all textile materials. The scalability of the technology was demonstrated (Figure 3) through the application of the substance using either a spray or a brush. The technology was found to be applicable to real-world objects and permitted the hydrophobising of garments constructed from diverse materials [95].

**Figure 4:**
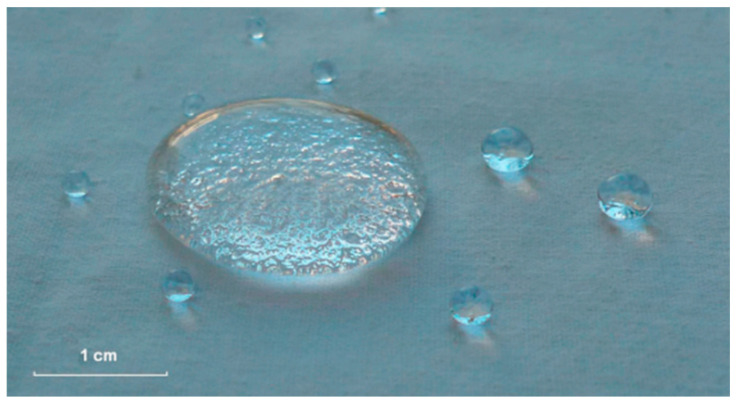
Water droplets on chitosan-modified cotton textile material [96].

N. Ivanova and A. Philipchenko have proposed a method of surface modification of cotton materials using cross-linked chitosan nanoparticles. The preparation of the chitosan nanoparticles was achieved through a modification process of a solution of chitosan in dilute acetic acid, with the addition of the surfactant heptadecafluoro-1-octanesulfonate. This modification process was conducted under constant stirring for a duration of one hour. The basis of the particle interaction was the electrostatic interaction between the amino groups of the chitosan and the anionic group of the surfactant. The solution was then applied to the cotton surface by spraying, after which the material was washed and air dried on multiple times. The wetting edge angle of the modified material was found to be contingent on the number of anionic groups present within the molecule of the chitosan, which in turn exerted an influence on the charge of the chitosan nanoparticle. Consequently, the content of 0.8 to 1 anionic group per 1 elementary unit of chitosan provides an edge wetting angle in the region of 156–157° (Figure 4). Simultaneously, the coating, which contained 0.9 fluoro-groups per elementary unit of the polysaccharide chitosan, exhibited elevated stability of the wetting angle over a period of 30 h. It is important to note that this coating demonstrated resilience to 10 washing cycles of 30 min, exhibiting only a slight change in wetting angle of 1–2° [96].

## 4. Non-Combustible (Flame-Retardant) Textiles

Combustion has thus far been defined as a self-accelerating exothermic redox process accompanied by glow and flame formation. Uncontrolled combustion has the potential to cause significant material damage and adverse effects on human health. The creation of non-combustible textiles or materials that can limit the spread of fire can significantly improve safety in the event of fires and flames.

The development of non-combustible textile structures has been a subject of interest to scientists for millennia [97,98]. High interest was ensured by increasing safety and preserving human life and property [99,100]. To create non-combustible materials can be used modification during the production process, in which chemical modification of molecules (e.g., copolymerisation) or creation of hybrid materials, in which substances or materials are integrated into the textile matrix. In addition, post-production modification of the material can be used, whereby modifying compositions can be applied to the surface of the textile. Inorganic substances can be used for modification (both during production and post-production) [101], polymers [102], nanoparticles [103,104], biomacromolecules [105,106] as well as their combinations and hybrids [107,108]. It should be noted that flame retardants are not designed to prevent the material ignition, but rather to limit the rate of flame spread, to prevent sustained combustion and to increase the ignition conditions of materials. Furthermore, flame retardants release safe substances and gases that serve to limit combustion.

### 4.1. Methods for Controlling the Flammability of Textile Materials

There are currently various approaches to analysing the flammability and combustibility of textile materials. Many textile materials have special standards that may vary depending on the country and the material’s intended use. Modern physico-chemical analysis methods, such as IR spectroscopy and TGA, can also be employed. It is also relevant to develop new methods that would allow for a comprehensive assessment of the flammability of Gorenje textile materials. Table 1 shows the main approaches that can be used to analyse the combustion processes and fire resistance of textile materials.

The amalgamation of methodologies enables comprehensive oversight of the fire resistance properties of materials. Modern methods are relatively straightforward and enable precise evaluation of the flammability and fire resistance of materials, as well as composite textiles. Furthermore, the methods outlined in the table facilitate a comprehensive comparison of samples modified with different formulations, or even an assessment of fire resistance between materials of different origin (synthetic, natural).

### 4.2. Traditional Methods of Creating Fire-Resistant Textiles

One of the conventional methods of imparting fire resistance is the utilisation of antimony compounds [109]. The utilisation of SbO_3_, Na_3_SbO_4_, and other compounds has been demonstrated to be effective in the context of fire resistance. The mechanism of action is related to the catalytic action of antimony and the acceleration of carbon oxidation. Furthermore, the combination of antimony compounds with halogens has been demonstrated to result in the formation of halides, which have been shown to impede the propagation of flames.

An example of the use of antimony oxides to create flame-retardant agents for textiles is the work of Hyelim Kim et al. [110]. In this work, antimony trioxide and tetroxide were added to a poly (acrylonitrile-co-vinylidene chloride) copolymer matrix. The polymer fibres were obtained using a wet spinning system where dimethyl sulphoxide (DMSO) was used as solvent.

In recent years, the utilisation of antimony compounds has been subject to significant limitations, owing to the revelation that antimony trioxide possesses the capacity to impede carbon oxidation [111]. This phenomenon has led to a substantial diminution in the efficacy of such substances devoid of halogen elements. Simultaneously, antimony-based flame retardants entailed a substantial financial expense, thereby constraining the large-scale industrial utilisation of such compounds. The incorporation of antimony oxides into the material has been shown to enhance its flame-retardant properties, with a significant increase in the LOI (limiting oxygen index) from 26% to 31.2%.

Furthermore, the use of boron compounds as flame retardants for textile materials has gained significant traction. It is hypothesised that borax (Na_2_B_4_O_7_), boric acid, zinc borate, ammonium fluoroborate (NH_4_BF), and other substances, could be utilised as potential modification agents. It has been demonstrated that boron compounds are capable of facilitating the cross-linking of polymer materials during the pyrolysis of textiles. This process has the potential to reduce the rate of decomposition and the subsequent release of volatile combustibles into the atmosphere. It has been demonstrated that certain boron-based substances have the capacity to form esters with cellulosic material. The compound exhibited a glassy structure, which resulted in the formation of a carbon coating during the combustion process. This prevented solid-phase oxidation of carbon.

An example of the use of boron-based systems is the work of Kongliang Xie et al. [112]. Cotton was soaked in an aqueous solution of boric acid and 2,4,6-tri[(2-hydroxy-3-trimethylammonium)propyl]-1,3,5-triazine chloride, by double dipping and spinning, and then dried at 95 °C for 3 min and fixed the composition at 165 °C also for 3 min. The influence of boron-containing substances in flame-retardant compositions for fabrics is comprehensively examined in separate articles [113].

The use of certain boron compounds, such as zinc borate, has been demonstrated to enhance the insolubility of materials in water. The application of organic solvents has been identified as a key challenge in the utilisation of such materials for textile applications. This challenge arises from the complexity of the application process and the resultant increase in economic costs. Furthermore, treatment with boron compounds has been demonstrated to result in substantial alterations to the mechanical properties of textile materials, in addition to a reduction in air permeability. This is a property that is considered unacceptable for certain types of textile materials. Many materials used in clothing production have a few requirements regarding mechanical properties and air permeability, which is important for body thermoregulation. Therefore, significant changes in these properties will not allow for the use of such compositions as a basis for functionalization. It is important to note that all of the above increases the prospects for the use of boron compounds to improve the fire resistance of organic materials, including plastics.

Furthermore, the utilisation of inorganic and organic phosphorus compounds has been employed in the fabrication of flame-retardant textile materials. Ammonium polyphosphates, ammonium sulphamate, phosphate esters, phosphonium derivatives and phosphonates can be used as flame retardants. These compounds find active application in the modification of cellulosic textile materials, as well as polymeric materials, where they function as foaming agents. Phosphorus compounds have been demonstrated to possess the capacity to impart long-term flame retardancy, in addition to enhancing the mechanical properties of textile materials.

As demonstrated by Sabyasachi Gaan and Gang Sun, the use of phosphorus-based substances as flame-retardant agents for textiles is well-documented [114]. The application of flame-retardant formulations to cotton was achieved through the use of aqueous or acetone-based solutions, depending on solubility. Fabric samples were then passed through a laboratory pulveriser to control wettability. Subsequently, the fabrics were subjected to an incubation process at a temperature of 80 °C for a duration of 10 min. This was followed by a period of incubation at ambient temperature for a duration of 24 h.

The utilisation of organophosphorus compounds as additives in bulk and as surface modifiers for textile materials of various natures is a consequence of their unique flame-retardant properties and large chemical diversity.

### 4.3. Modern Methods of Creating Fire-Resistant Textiles

**Figure 5:**
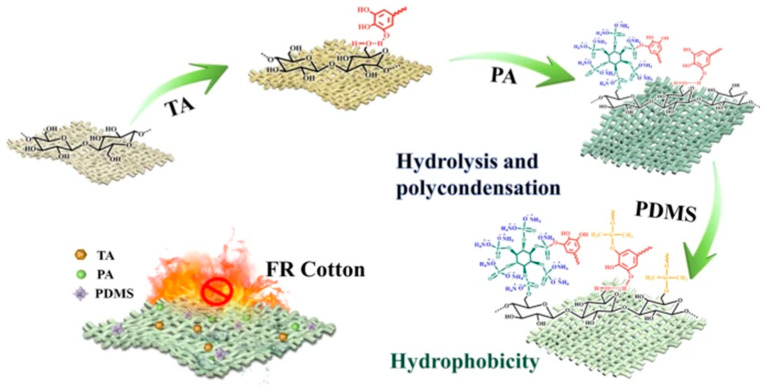
Scheme of modification of textile materials [115].

A significant number of contemporary developments continue to utilise phosphorus residues or phosphorus-containing acids in the production of flame retardants for textiles. Meini Yang advanced a methodology for the production of non-combustible cotton fabric. For the purpose of this study, an acidified aqueous solution of tannic acid containing sodium lauryl sulphate at a concentration of 0.25% was prepared. The fabric was then soaked in this solution, after which it was immersed in a solution of carbamide and phytic acid (PA) at varying concentrations for a period of 30 min. Following this, the samples were dried and immersed in a PDMS (polymethylsiloxane) solution (in tetrahydrofuran) that had been treated with ultrasonic waves for a duration of one hour. The samples were then dried in an oven at a temperature of 80 °C for a period of 30 min (Figure 5). The efficacy of the three-step treatment was substantiated by the identification of characteristic infrared spectroscopy peaks for each of the modifying agents and by the elemental analysis of the modified textile material. The extent of fabric degradation was found to be contingent upon the concentration of phytic acid within the system. At a concentration of 8%, the damage was measured at 60 mm, while untreated cotton exhibited complete combustion (150 mm). Tests with cone colorimetry demonstrated that the total heat release of the material modified with a solution containing 8% phytic acid was reduced by half, smoke generation was decreased from 1.8 to 1.0 m^2^, and the residual carbon content increased by 12%. These findings suggest that the coating exhibited a catalytic carbonisation effect, with the formation of a protective carbon layer. The self-extinguishing properties of the material were demonstrated even after 40 washing cycles, which demonstrates the high stability of the coating to external influences [115].

The work by X.-W. Cheng et al. also examined a phytic acid-based flame retardant, which showed high potential for improving the flame retardancy of silk fabrics. The material demonstrated a significant increase in LOI and was also relatively easy to apply to textiles. Such environmentally friendly methods could accelerate technology transfer to enterprises and ensure the safety and environmental friendliness of functionalization processes [116].

**Figure 6:**
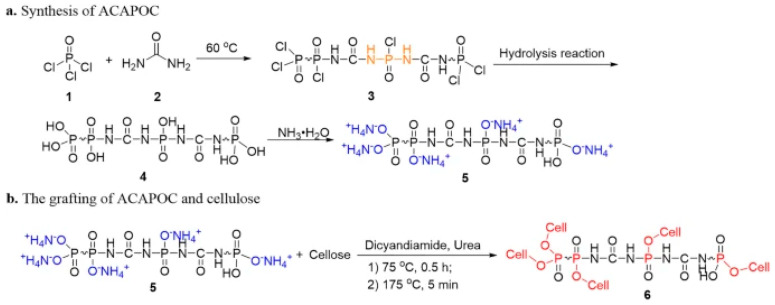
Scheme of ACAPOC flame-retardant production and cotton modification [117].

In their study, Kunling Liu et al. developed a method for the preparation of a flame-retardant agent based on carbamide and phosphorus trichloride, which was used to protect cotton textile materials. Phosphorus oxychloride and carbamide were utilised as precursors for the synthesis, with the polymeric flame-retardant agent ACAPOC (polymeric flame retardant) being obtained in three steps (Figure 6). The application of the formulation to cotton was achieved through the process of soaking the fabric in a thermostatic oscillator at a temperature of 75 °C for a duration of 30 min. Additionally, the introduction of 5% dicyandiamide and 10% carbamide into the system served as catalysts. Following the conclusion of the treatment process, the tissues were subjected to a drying procedure at a temperature of 75 °C for a duration of five minutes. This was followed by a washing step to ensure the removal of any residual substances. In this study, compositions with varying ACAPOC content were utilised within the system. Treatment with a modifying compound with a mass content of 25% resulted in a significant enhancement in the thermal stability of the materials when subjected to heating in nitrogen and in air. A comparative analysis of treated and untreated fabric was conducted utilising cone calorimetry. The findings revealed that the time to ignition (TTI) of the native samples was 22 s. In contrast, the samples with ACAPOC exhibited no TTI and retained an intact carbon skeleton structure. It is important to note that the maximum (PHRR) and total THR heat release rate decreased by 90.8% and 61.8% after cotton modification. This approach led to a substantial reduction in the propagation of fire, which can significantly enhance the safety of the material and mitigate damage in the event of an ignition. It is important to note that the effectiveness of the flame-retardant effect was maintained even after 50 washes [117].

**Figure 7:**
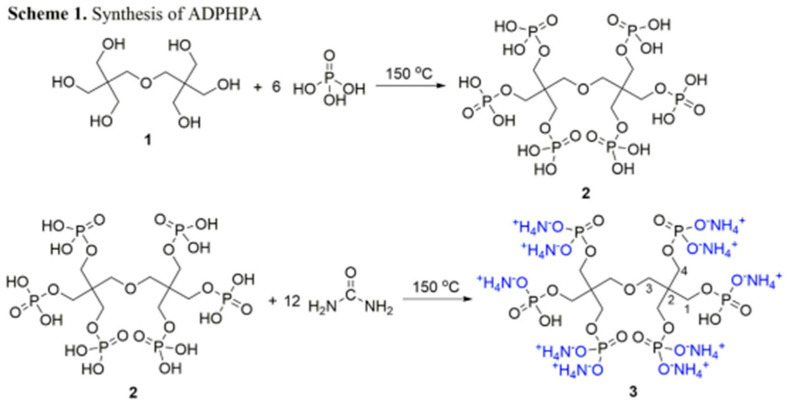
Schematic diagram of ADPHPA production [118].

This team of authors also developed a flame-retardant formulation based on dipentaerythritol (Di-PE) and phosphoric acid with the addition of carbamide [118]. A two-step process resulted in the flame-retardant agent ADPHPA (ammonium salt of dipentaerythritol hexaphosphoric acid). As illustrated in Figure 7, the agent in question also yielded a zero TTI value, in contrast to the untreated sample, which exhibited a TTI value of 11 s. The spread of fire in cotton treated with the 30% solution was reduced by a factor of 20, which markedly increases the material safety of the treated formulations. The surface modification demonstrated durability, withstanding 50 wash cycles and maintaining its resistance to fire. The limiting oxygen index (LOI) was determined to be between 44 and 49% in the modified but not washed fabric and between 27 and 37% in the modified and washed materials. These figures exceed the critical value of 26%, thus classifying the material as a flame retardant. In this instance, the modification led to a decline in cotton strength, from 26 to 35%, attributable to the formation of bonds with ADPHPA and concurrent breakage of intermolecular and Van der Waals bonds, resulting in a reduction in strength.

Gamal Zain proposed a similar mechanism for the covalent modification of cotton textile surfaces using polyphosphorous compounds. A multi-stage cotton modification process was proposed, which, when chitosan was added to the modifying composition, demonstrated high fire-resistant properties that provided up to 95% of the residual mass during the vertical flame test [119].

**Figure 8:**
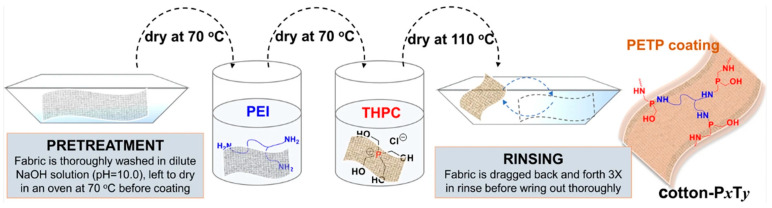
Scheme for creating fire-resistant fabrics [120].

Bin Zhao et al. proposed a method of modifying cotton in an aqueous solution using branched polyethylenimine (PEI) and tetrakis(hydroxymethyl)phosphonium chloride (THPC). This process involved a Mannich-type reaction on the surface of the textiles, resulting in the formation of poly[ethylenimine-tris(dimethylhydroxyethyl)phosphine] (PETP), which imparted a fire-resistant coating to the textiles. The modification was carried out in two stages, with different precursor contents used in each stage, and the textiles were dried after each stage (Figure 8). The success of the modification was confirmed using infrared spectroscopy. Using 10% PEI and 1% THPC prevented the material from burning and resulted in charring of the cotton. Increasing the THPC content from 5% to 10% did not significantly affect the fire resistance or thermal stability. The treated material retains up to 97% of its mass during vertical flame testing and only experiences slight charring. Following a single wash cycle, the material exhibits no reduction in fire resistance; however, subsequent to the second wash cycle, the material is extensively charred. Concurrently, the LOI for processed materials ranged from 29 to 30%, thereby substantiating the elevated fire resistance properties of textile materials. Furthermore, the processing of the material has been shown to result in a decrease in the maximum and total heat release rate by a factor of five in comparison with unprocessed cotton. Utilising TG-FTIR analysis, it was determined that the modification facilitates an augmentation in the quantity of non-flammable volatile substances during the pyrolysis of processed textiles. It is imperative to ensure that toxic phosphorus gas compounds are not detected, thereby guaranteeing the safety of the material’s thermal decomposition and enabling the utilisation of PETP as a prospective agent for the protection of textile materials against fire [120].

**Figure 9:**
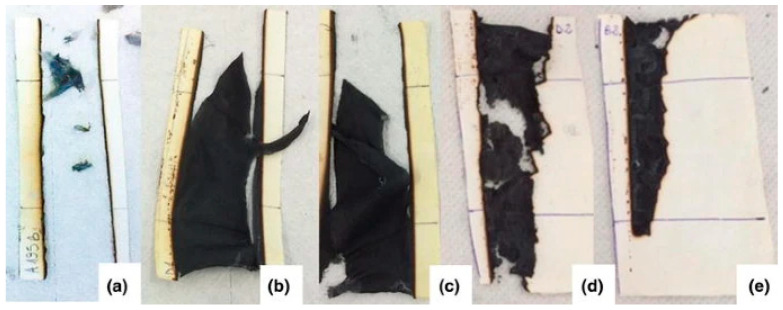
HFT for: (**a**) cotton, (**b**) cotton + TACR/WP (4 layers); (**c**) cotton + TACR/WP (8 layers); (**d**) cotton + TiO_2_CIT/casein (4 layers) and (**e**) cotton + TiO_2_CIT/casein (8 layers) [121].

A flame-retardant modifying compound based on titanium dioxide nanoparticles with proteins adsorbed on them was presented by Simona Ortelli et al. The study evaluated the effects of different types of protein (whey or casein) as adsorption agents on the surface of the nanoparticles, which were used as flame retardants in tissue. To modify the proteins, whey proteins were stabilised with alkali and caseins with sodium citrate, after which they were mixed with the nanoparticles at a TiO_2_:protein ratio of 0.7:1. Coupling occurred between the particles and proteins due to the particles’ high specific surface area and electrostatic interaction. The particles with adsorbed proteins were then suspended in water and used to impart fire-resistant properties to textiles. The modifying compound was applied to cotton fabrics via immersion for three minutes, followed by padding and drying at 100 °C, then curing at 130 °C for 10 min. The proteins adsorbed on the particles exhibited high stability at four and eight coating layers, remaining intact and not desorbing into the liquid medium. The horizontal flame test (HFT) showed that native tissue burned completely without residue when exposed to a 3 s methane flame, while the time and area of combustion depended on the composition of the modifier, the number of deposited layers, and the type of proteins adsorbed onto the nanoparticles (Figure 9). The most effective system included nanoparticles coated with casein (with sodium citrate in the system), applied in eight layers to cotton material. Cone colorimetry data showed that the rate and maximum heat release decreased even when treated with four layers of the modifying compound containing any proteins. Cotton material coated with eight layers of casein particles did not ignite, indicating a significant improvement in fire resistance [121].

Nanoparticles of O-carboxymethyl chitosan (CT-O) can be used to create flame-resistant materials. A fire-resistant composition based on CT-O decorated with graphene is proposed in work published at [122].

This material was synthesised in a chitosan solution containing propanol and sodium hydroxide. After stirring, monochloroacetic acid was added to the solution and the resulting precipitate was filtered, washed and dried at 30 °C. The solution was then mixed with graphite and subjected to ultrasonic treatment for 60 min before being separated from the solution by centrifugation. Textiles were immersed in a suspension of O-carboxymethyl chitosan decorated with graphene in order to impart fire-resistant properties. Additionally, the material could be modified with silver nanoparticles and polystyrene through impregnation with a silver precursor and the subsequent formation of polypyrrole (Ppy). This double modification (CT-O/G and AgNPs and Ppy) increased the thermal stability of the textile material while achieving a maximum coal yield of 13.3% during pyrolysis. The burning rate of the treated fabric decreased from 150 mm/min to 31 mm/min, demonstrating the material’s exceptional fire-resistant properties, and its LOI reached 22.8%, meaning that it can be classified as fire-resistant [122].

Qingqing Zhou et al. presented a modification of cotton fabric involving holding it in a polyethylenimine (PEI) solution (along with crosslinking), drying it, immersing it in a phytic acid (PA) solution and drying it again at 80 °C. The fabric was then held for two minutes at 120 °C for fastening. The samples were then neutralised and dried again. The PEI-PA coating formed a transparent film on the surface and homogeneous bubbles, resulting in foam coke formation. This foam coke can act as a heat insulator and smoke suppressor. This layer limited combustion, prevented heat from penetrating the condensed phase and limited the supply of oxygen to the combustion zone. The LOI of the modified material was 37%, which is significantly higher than that of the untreated material (18%). It is important to note that the fire resistance of modified cotton samples is unaffected by up to 20 washing cycles [123].

**Figure 10:**
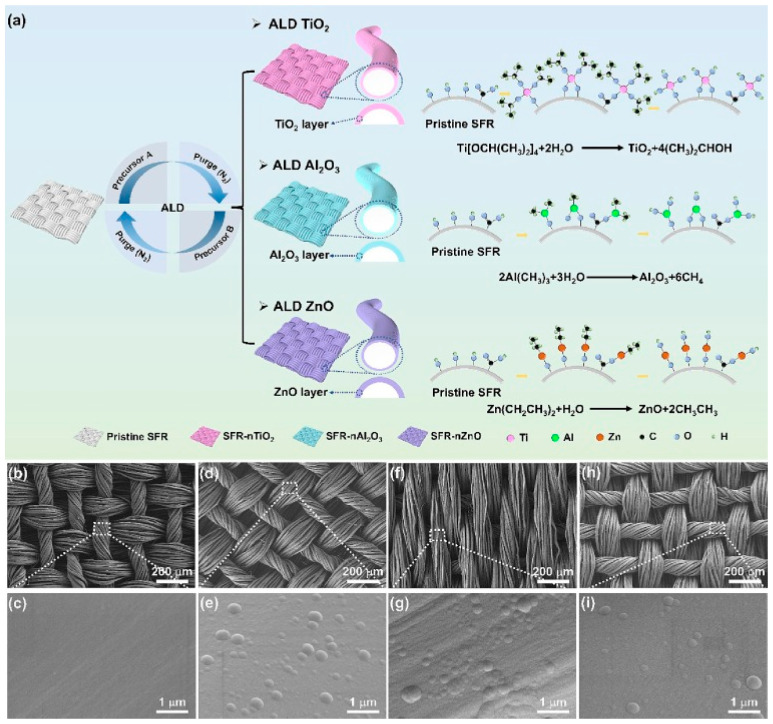
(**a**) The strategy of applying metal oxides to the surface of a textile material, the morphology of the textile surface before (**b**,**c**) and after modification (**d**–**i**) by atomic layer deposition [124].

A method for increasing the fire resistance of silk materials by atomic layer deposition of metal oxides on the surface was presented by Yu Zhang and the rest of the team. Titanium dioxide (TiO_2_), aluminium oxide (Al_2_O_3_) and zinc oxide (ZnO) were applied to the surface of the tissues in several layers, with a total thickness of no more than 200 nm (Figure 10). This process took place in a specialised installation in which the metal precursors were in the form of alkoxides, water was the oxygen source and nitrogen was the carrier gas. The successful application of the layers was confirmed using XPS and XRD. Vertical combustion tests showed that all modified compounds effectively maintained the integrity of the material’s structure, unlike pure silk, which completely burned in just 7 s. This underscores the inherent flammability of silk, a natural protein fibre. Notably, no secondary flame formation was observed on samples coated with the modifying compound during testing, a critical safety aspect as it indicates a lower risk of fire spread. Among the tested samples, zinc-coated samples demonstrated the shortest burnt zone length, demonstrating the effectiveness of the modification in improving fire resistance. Such advances in flame-retardant treatments are crucial for applications in the textile industry, particularly in safety-sensitive industries such as apparel and home furnishings. The LOI of the materials depended directly on the number of layers applied to their surfaces. At the same time, it increased from 24% to 28% in zinc-modified samples. Calorimetric tests showed that pure silk fabrics burned completely when exposed to radiation (25 and 50 kW/m^2^), while samples modified with metal oxides retained their original shape in both cases. It is important to note that atomic layer deposition did not affect the mechanical properties or air and moisture permeability. This makes it easy to integrate these materials into various types of clothing and other products [124].

It is important to note that this method is expensive and difficult to scale up, which can make it hard to use in real technological schemes and limit its integration. However, despite the cost and complexity of the production process, this technology can be used to create specialised fabrics with high breathability and fire resistance requirements.

## 5. Prospects and Main Limitations

### 5.1. Multifunctionality

The creation of multifunctional compounds has helped integrate functionalization into the textile industry. Single-stage fabric processing that allows creating simultaneously hydrophobic and non-flammable materials would reduce the cost of production processes and reduce the number of technological operations. At the same time, multifunctional compounds would be of great importance for personal protective equipment and specialised clothing.

### 5.2. Improving the Environmental Friendliness of the Functionalization Process

The creation of natural modifiers for fabric would actualize their use and also improve the environmental friendliness of the enterprise. The use of aqueous solutions and suspensions instead of organic solvents for hydrophobization, as well as the use of organic acids instead of antimony compounds for non-flammable creation of non-flammable can significantly reduce the impact of such industries on the environmental state of the territories where they are located. This can reduce the costs of wastewater treatment, air, personal protective equipment for employees and taxes that enterprises pay for the use of toxic agents for modification.

### 5.3. Maintaining the Basic Properties of Fabric

High-molecular substances and complex systems based on them can be used as potential agents for modification. The use of polymers can significantly change the morphology of the fabric, as well as reduce the air permeability of the native material, which together will significantly affect the comfort of using products. A potential solution may be a significant reduction in high-molecular substances in modifying compositions, as well as the use of low-molecular substances and nanomaterials. In addition, the use of such materials will significantly increase the stability of modifying systems, as well as simplify the processes of washing and recycling.

### 5.4. Establishing the Effect of Functionalization on Colorimetric Characteristics

Our review article presents promising systems for the functionalization of various textile materials. When purchasing clothing and textiles, consumers primarily consider the aesthetic properties of the product. Therefore, the interaction between modifying agents and dyes is becoming an important stage in modern research. At the same time, applying the composition to the surface, keeping it in modifying compositions, washing and drying can dramatically affect the colour and brightness of the fabric. But at present there are practically no works establishing the relationship between the modification conditions and the colorimetric characteristics of the final textile product. Increasing the number of such works for fabrics of various nature could improve the procedures for the functionalization of fabrics, as well as integrate them into industry.

### 5.5. Study of Scaling Processes

At present, the proposed methods reflect a set of standard laboratory operations that allow successfully creating hydrophobic and non-flammable textile materials. But given the volumes of textile production, it is necessary to confirm the possibility of using modifying processes for large volumes of materials. For this, installations for large volumes of fabric should be developed, and the dependence of the amount of material and the success of material functionalization should be studied. Such works could serve as a basis for including functionalization stages in the main technological processes of clothing production.

## 6. Conclusions

In this review, we systemise various strategies for the functionalization of textile materials, including hydrophobic, flame-retardant, and antibacterial treatments, as well as post-production modification methods. Strategies for tuning the surface morphology and chemical modification that can be integrated to create hydrophobic clothing are presented. The presented strategies allow the wettability of the material to be controlled without violating the colorimetric characteristics, as well as having a minimal impact on the mechanical properties and strength. The main trends in the creation of fire-resistant textiles are analysed, and the possibility of manufacturing such products using natural agents is shown, which will increase the environmental friendliness of the modification process.

It is important to note that most of the products we highlighted utilise simple application methods, allowing them to be modified at various stages of garment production. These approaches can serve as a basis for both modifying everyday clothing and creating personal protective equipment. Comprehensive studies evaluating the impact of processing conditions on properties responsible for garment comfort (breathability and mechanical properties) and aesthetics, as well as studies evaluating the transfer of functionalization technology from laboratory to industrial scale, have helped integrate modification processes into the textile industry.

## Figures and Tables

**Table 1:** Methods of analysing the fire resistance and flammability of textile materials.

№	Name	Method Description	Controlled Variable	Notes
1	Horizontal flame test (HFT)	The essence of the method is to ignite the fabric in a horizontal configuration for some time (usually 12 s).	The time of the residual flame, the speed of flame propagation, the mass of the residue and the length of charring can additionally be analysed.	There is a standard ASTM D4986, etc.
2	Vertical flame test (VFT)	The essence of the method is to ignite the fabric in a vertical configuration for some time (usually 12 s).	The time it takes for the flame to travel a certain distance is controlled, and the mass of the residue and the length of charring can additionally be analysed.	There is a standard ASTM D 6413-08, ASTM D6413-99, etc.
3	Limiting oxygen index (LOI)	The test is conducted within a specialised atmospheric chamber, where the oxygen level is gradually reduced until the flame on the material is extinguished.	The minimum oxygen content in the atmosphere required to sustain combustion of the material is 21%. This indicator is related to the oxygen content in the atmosphere. If it is higher than 21%, then the material is considered to have increased fire resistance.	There is a standard ISO 4589:1996, ASTM D 2863, ASTM D2863-2000, etc.
4	Cone calorimeter and Micro cone calorimeter (MCC)	This method involves measuring the rate at which heat is released when materials burn. The consumption of oxygen during the combustion process is what this method is related to.	Maximum heat release rate (PHRR), heat release rate (HRR) and total heat release (THR), fire propagation rate (FGR), etc.	There is a standard ISO 5660-1, ASTM D 7309, etc.
5	The 45° Angle Test	The essence of the method is to ignite a tissue sample, which is held at an angle of 45° in a ventilated chamber.	The flame propagation time, including ignition, is measured.	There is a standard AATCC 1933–1962
6	Thermogravimetric (TG) analysis	The method is based on controlled heating of textile material in various atmospheres (air, oxygen, nitrogen and argon are usually used)	It allows you to establish the thermal stability of a textile material, as well as to estimate the mass amounts of material loss at various temperatures.	Special equipment is required.
7	TG-FTIR analysis	The method is predicated on the detection of infrared spectra at a range of temperatures.	The apparatus provides control of groups and valence bonds, which can be used to analyse released substances during the heating of textile material.	Special equipment is required.
8	Smoke Test	The fundamental principle of the method involves the vertical fixation of the sample, followed by its exposure to flame at a distance of no more than 2.5 cm.	The density of the smoke released is measured. Photometric methods have been employed to regulate this phenomenon.	
9	The Metal Cylinder	The fundamental principle of the method is predicated on the placement of a steel cylinder, which is heated to a temperature of 800 °C for a duration of 12 s.	A rigorous experimental process is undertaken to ascertain the combustion time and charring area of the textile material.	This method can be considered relatively exotic. The material is utilised in the manufacture of specialised protective equipment, carpets and outerwear.

## Data Availability

No new data were created or analysed in this study. Data sharing is not applicable to this article.
